# Developing a systems-based framework of the factors influencing dietary and physical activity behaviours in ethnic minority populations living in Europe - a DEDIPAC study

**DOI:** 10.1186/s12966-017-0608-6

**Published:** 2017-11-07

**Authors:** Michelle Holdsworth, Mary Nicolaou, Lars Jørun Langøien, Hibbah Araba Osei-Kwasi, Sebastien F. M. Chastin, F. Marijn Stok, Laura Capranica, Nanna Lien, Laura Terragni, Pablo Monsivais, Mario Mazzocchi, Lea Maes, Gun Roos, Caroline Mejean, Katie Powell, Karien Stronks

**Affiliations:** 1 Public Health Section, School of Health and Related Research-ScHARR, The University of Sheffield, Sheffield, UK; 2 Academic Medical Centre, University of Amsterdam, Department of Public Health, Amsterdam Public Health research Institute, Amsterdam, The Netherlands; 3 Department of Physical Education, Norwegian School of Sport Sciences, Oslo, Norway; 4 Institute for Applied Health Research, School of Health and Life Science, Glasgow Caledonian University, Glasgow, UK; 5 Department of Psychological Assessment and Health Psychology, University of Konstanz, Constance, Germany; 6 Department of Movement, Human and Health Sciences, University of Rome Foro Italico, Rome, Italy; 7 Department of Nutrition, University of Oslo, Oslo, Norway; 8 Department of Nursing and Health Promotion Faculty of Health Sciences, Oslo and Akershus University College of Applied Sciences, Oslo, Norway; 9 UKCRC Centre for Diet and Activity Research, MRC Epidemiology Unit, University of Cambridge School of Clinical Medicine, Box 285, Institute of Metabolic Science, Cambridge Biomedical Campus, Cambridge, CB2 0QQ UK; 10 Present address: Department of Nutrition and Exercise Physiology, Elson S Floyd College of Medicine, Washington State University, Spokane, WA 99210-1495 USA; 11 Department of Public Health, Ghent University, Ghent, Belgium; 12 Department of Public Health, Ghent University, Ghent, Belgium; 13 Consumption Research Norway SIFO, Oslo and Akershus University College of Applied Sciences, Oslo, Norway; 14 UMR MOISA, Campus INRA-SupAgro de la Gaillarde, Montpellier, France

**Keywords:** Minority populations, Europe, Migrants, Immigrants, Physical activity, Sedentary behaviour, Diet, Framework, Systems

## Abstract

**Background:**

Some ethnic minority populations have a higher risk of non-communicable diseases than the majority European population. Diet and physical activity behaviours contribute to this risk, shaped by a system of inter-related factors. This study mapped a systems-based framework of the factors influencing dietary and physical activity behaviours in ethnic minority populations living in Europe, to inform research prioritisation and intervention development.

**Methods:**

A concept mapping approach guided by systems thinking was used: i. Preparation (protocol and terminology); ii. Generating a list of factors influencing dietary and physical activity behaviours in ethnic minority populations living in Europe from evidence (systematic mapping reviews) and ‘eminence’ (89 participants from 24 academic disciplines via brainstorming, an international symposium and expert review) and; iii. Seeking consensus on structuring, rating and clustering factors, based on how they relate to each other; and iv. Interpreting/utilising the framework for research and interventions. Similar steps were undertaken for frameworks developed for the majority European population.

**Results:**

Seven distinct clusters emerged for dietary behaviour (containing 85 factors) and 8 for physical activity behaviours (containing 183 factors). Four clusters were similar across behaviours: Social and cultural environment; Social and material resources; Psychosocial; and Migration context. Similar clusters of factors emerged in the frameworks for diet and physical activity behaviours of the majority European population, except for ‘migration context’. The importance of factors across all clusters was acknowledged, but their relative importance differed for ethnic minority populations compared with the majority population.

**Conclusions:**

This systems-based framework integrates evidence from both expert opinion and published literature, to map the factors influencing dietary and physical activity behaviours in ethnic minority groups. Our findings illustrate that innovative research and complex interventions need to be developed that are sensitive to the needs of ethnic minority populations. A systems approach that encompasses the complexity of the inter-related factors that drive behaviours may inform a more holistic public health paradigm to more effectively reach ethnic minorities living in Europe, as well as the majority host population.

**Electronic supplementary material:**

The online version of this article (10.1186/s12966-017-0608-6) contains supplementary material, which is available to authorized users.

## Background

Some ethnic minority groups living in Europe have a high prevalence of preventable non-communicable diseases (NCDs), such as obesity, type 2 diabetes and cardiovascular diseases [1–4]. Diet and physical activity behaviours are likely to play a role in their aetiology and differences in these behaviours compared with host populations are well documented [4–10]. However, there are fewer studies of the differences in the underlying factors influencing these behaviours [11–13]. Most studies have focused either on a small number of minority groups or are limited to specific European countries, which makes it challenging to generalise about the nature of any differences [14–20]. It could be argued that the variation between ethnic groups is as great as that between the general European population. Nonetheless, it could be expected that there are commonalities in people’s lives who are either first or second generation migrants, which are distinct from the general population [11–13]. Thus, understanding the factors underlying diet and physical activity behaviours in ethnic minority groups is a first step to informing the development of public health interventions that are successful in reaching minority ethnic populations in Europe.

Existing frameworks are insufficient for prioritising research or interventions development, as they either focus on migration and dietary acculturation processes [13] or on a specific health outcome, such as obesity in the whole population [21, 22]. Alternatively, they are based on evidence of ethnic minority populations living outside of Europe, for example, of African descent in the US [21] or Iranians living in Australia [23]. This may offer useful insights, even though contextual differences limit their transferability to Europe. Likewise, focusing only on obesity has the potential to ignore important drivers of the complex system of factors influencing dietary and physical activity behaviours.

A system-based approach has the potential to cast a holistic analytic lens [24] to developing interventions, because it is based on the interrelationship of clusters within a dynamic system. Systems thinking can simply be defined as *‘looking at things in terms of the bigger picture’* [25]. Dietary and physical activity behaviours therefore emerge as a property, which cannot be resolved from simple, uni-faceted interventions [26]. Shifts are likely to be required within multiple clusters of factors, even though some of these may only have small effects on individuals, they have the potential to stimulate population changes when combined [26]. Therefore, the aim of the current study was to develop a systems-based framework of the factors influencing dietary and physical activity behaviours in ethnic minority populations living in Europe, to be able to inform research prioritisation and the development of interventions to reach these groups.

## Methods

The framework was constructed as part of the DEDIPAC-KH (DEterminants of DIet and Physical ACtivity Knowledge Hub) [27] for European populations. Within the DEDIPAC-KH, an inter-disciplinary group focused on the determinants of dietary and physical activity/sedentary behaviours. The task was undertaken at several steps involving scholars with varying academic backgrounds and from different countries (Table 1 for more details).

**Table 1:** Characteristics of participants in the different concept mapping stages to develop the framework

	Stage i, ii: Preparation and generation of factors	Stage iii: Structuring, rating and producing cluster map	Stage iv: Interpretation and utilisation
Participant numbers	*n* = 12 ethnic minorities DEDIPAC team *n* = 21 DEDIPAC general population team	*n* = 12 ethnic minorities DEDIPAC team *n* = 21 DEDIPAC general population team	International symposium: *n* = 44 delegates and *n* = 2 invited experts Finalisation *n* = 18 members of DEDIPAC ethnic minorities and general population teams
Fields of expertise	Agricultural economics Behavioural economics Behavioural nutrition Consumer science Dietary inequalities Dietetics Epidemiology Exercise physiology Health inequalities Medicine Migrant health Nutritional epidemiology Physical activity Psychology (health, cognitive and social) Public health nutrition Social anthropology Social demography Social inequalities Sociology of health	Agricultural economics Behavioural economics Behavioural nutrition Consumer science Dietary inequalities Dietetics Epidemiology Exercise physiology Health inequalities Medicine Migrant health Nutritional epidemiology Physical activity Psychology (health, cognitive and social) Public health nutrition Social anthropology Social demography Social inequalities Sociology of health	Agricultural economics Behavioural economics Behavioural nutrition Behavioural sciences Consumer science Dietary inequalities Dietetics Epidemiology Exercise physiology Food engineering Health inequalities Medicine Migrant health Nutritional epidemiology Nutrition science Physical activity Physical education and physiotherapy Physical anthropology Psychology (health, cognitive and social) Public health nutrition Social anthropology Social demography Social inequalities Sociology of health
Countries	Belgium France Germany Ireland Italy Netherlands Norway UK	Belgium France Germany Ireland Italy Netherlands Norway UK	Australia Belgium Denmark France Germany Ireland Italy Netherlands Norway Poland Spain Sweden UK

A total of 89 participants contributed to at least one step of the creation of the framework. This comprised a team focusing on the factors influencing the behaviours of ethnic minority populations (‘DEDIPAC ethnic minority team’). Other teams in the DEDIPAC-KH focussed on the factors influencing dietary [28] and physical activity/sedentary behaviours [29, 30] of the general European population (‘DEDIPAC general population team’). This approach followed earlier successful examples of multidisciplinary partnerships that comprehensively described the factors influencing obesity-related behaviours [31].

The method was guided by concept mapping; drawing on both quantitative [32], and qualitative [33] approaches. Traditionally, quantitative concept maps have been used in health research, yet the case for flexible use of concepts maps has been advocated for, requiring a less rigid and more qualitative approach [33, 34]. Concept mapping is influenced by systems thinking and involves gathering and analysing different types of data and integrating these with prior research/experience [35]. Concept mapping was selected because it can illustrate how people visualize relationships between concepts within a map [34] and it can be used for research prioritisation [30]. The approach was structured around the four main phases proposed in quantitative concept mapping [32], but a more flexible mixed methodology was employed [34]: i. preparation; ii. generation of factors; iii. Structuring and rating factors into clusters; and iv. interpretation and utilisation of the framework.

### Preparation (terminology, protocol)

**Fig. 1:**
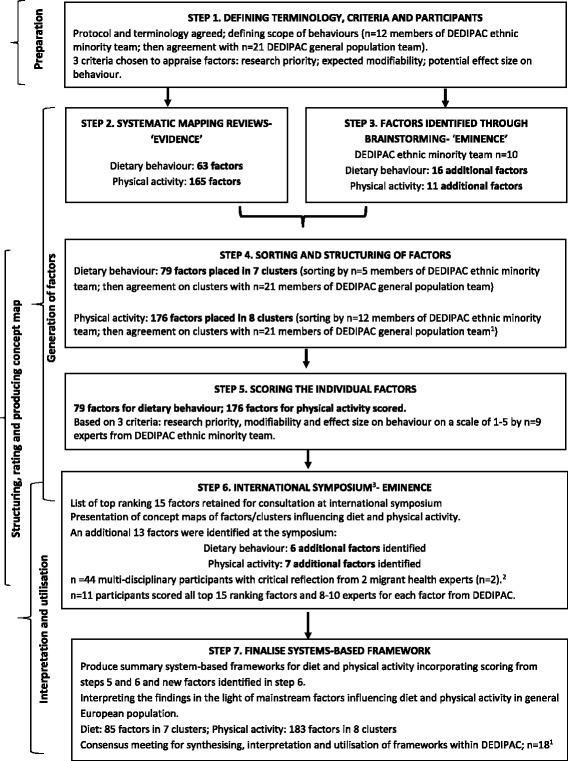
^1^Areas of expertise that were represented in the process are summarised in Table 1. ^2^Holdsworth M, Nicolaou M, Araba Saeed H, Jørun Langøien L, Powell K, Terragni L et al. (2015) Developing a framework map of the major determinants of dietary behaviour and physical activity/sedentary behaviour in minority ethnic groups living in Europe. International Society for Behavioral Nutrition and Physical Activity, June, Edinburgh (UK), abstract book p107, S6.73. https://eventmobi.com/api/events/7231/documents/download/596c6adb-ef0f-4695-be2c-8a1927877d2c.pdf/as/ISBNPA%202015%20bstract%20Book.pdf. Concept mapping process for developing the systems-based framework for dietary and physical activity behaviours in ethnic minority populations

The scope and purpose of the study protocol was developed (Fig. 1- step 1) by the DEDIPAC ethnic minority team, in consultation with the DEDIPAC general population team. Consensus was reached on the terminology for the different behaviours and for defining factors/correlates/determinants, so that there was a common understanding across the DEDIPAC-KH. Minority ethnic populations were defined as *‘immigrants/populations of immigrant background from low and middle income countries, population groups from the former Eastern Bloc countries who migrate to other parts of Europe and minority indigenous populations in Europe’*.

Three criteria were selected to score each factor, for its priority for future research (‘research priority’) and for how useful it would be in developing interventions (‘expected modifiability’; ‘potential effect size on behaviour’).

It was also decided how consultation outside of the DEDIPAC network could be undertaken, to ensure that a wide range of viewpoints were considered from diverse disciplines to encourage ‘buy in’ to the resulting framework [32]. The concept mapping process does not stipulate that all participants have to be involved in every step [32], so it was agreed that a small group would generate the factors and a larger group would sort and rate them.

### Generation of factors

The next phase (Fig. 1- steps 2 and 3; then steps 4 and 6) involved generating a set of factors to represent the entire conceptual domain of the topic area, i.e. the factors that influence diet and physical activity (incorporating sedentary behaviours) of ethnic minority populations living in Europe. This was conducted in parallel for diet and physical activity behaviours from published ‘evidence’ and expert opinion (‘eminence’). The aim was to develop wording that was detailed enough to convey the underlying meaning for each factor without requiring further explanation.

#### Published evidence (systematic mapping reviews)

Factors were generated from published ‘evidence’ by conducting systematic mapping reviews (Fig. 1- step 2) of the factors influencing dietary behaviours [36] and physical activity behaviours (incorporating sedentary behaviours) [37] among minority groups living in Europe. The review methods and protocols were published elsewhere [PROSPERO database #CRD42014013549/#CRD42014014575], but essentially primary data from quantitative and qualitative studies published over the 15 year period preceding data searching (1999–2014) were extracted. In synthesizing the findings, all of the factors (63 factors for diet; 165 factors for physical activity) were listed [36, 37]. Physical activity and sedentary behaviours were integrated together, as there was a lack of published research on sedentary behaviours of ethnic minority populations.

#### Expert opinion (‘eminence’)

Expert opinion was sought from three sources: brainstorming within the DEDIPAC ethnic minority team (Fig. 1- step 3) and by members of the DEDIPAC general population team (Fig. 1- step 4); and later by consultation with external experts during an international symposium (Fig. 1- step 6). Participants (*n* = 89 in total throughout the process) were from a range of disciplines (Table 1), but they were not all involved at every step.

Factors that had not emerged from the reviews, but could be important, were generated by the DEDIPAC ethnic minority team (Fig. 1- step 3). Existing frameworks were also used to extract additional factors [13, 21, 23, 38]. An additional 40 factors (to those from the systematic mapping reviews) were identified at steps 3 and 6 combined (22 for diet and 18 for physical activity).

### Structuring, rating and producing a concept map

#### Sorting and structuring of factors

The emerging factors from the systematic mapping reviews and expertise within the DEDIPAC-KH were grouped into clusters, according to how they were seen to relate to each other. This resulted in 7 dietary behaviour clusters containing 79 factors and 8 physical activity clusters containing 176 factors (Additional file 1: Tables S1 and S2). This process was undertaken in two ways (Fig. 1- step 4). Firstly, during meetings of the DEDIPAC ethnic minority team, when the relationships between factors were collectively debated. Secondly, during a confirmatory stage involving members of the DEDIPAC ethnic minority and the general population teams. The concept map that emerged was discussed collectively, which led to some changes in wording of the clusters to enhance clarity and some factors were moved into different clusters (Fig. 1- step 4).

#### Scoring the individual factors

All of the factors were scored individually (Fig. 1- step 5) by DEDIPAC ethnic minority team members and some general population team members (Table 1) using the three criteria identified in the preparation phase, i.e. ‘research priority’, ‘expected modifiability’ and ‘potential effect size on behaviour’. When scoring, individuals were asked to provide their scores based on their own professional judgments. The rating focus statement selected was: ‘Score the *following factors* for their importance on a scale of 1 to 5 for dietary behaviour [or physical activity and sedentary behaviour] in ethnic minority groups’, where 1 = the lowest importance and 5 = the highest. These three criteria were added together as a total measure of the perceived importance of individual factors for research and interventions. Individuals rated the factors separately and these scores were subsequently collated to develop the ranking of factors within clusters. The mean is the total sum divided by the number of factors, so a maximum of 15 could be gained for the three criteria on a Likert scale of 1–5, with 5 as most modifiable, strongest effect, largest priority. The position that the factors were ranked in was based on the overall sum divided by the number of factors in each cluster (Table 4).

#### International symposium- eminence

Consultation took place during a dedicated symposium at an international conference (Fig. 1- step 6) [39]. Symposium participants scored the top 15 factors that had emerged from step 5 (Fig. 1) using the same three criteria. The decision to only request scoring of the top 15 factors was for pragmatic reasons as the symposium was time limited. The symposium also allowed reflection on the next phase of ‘Interpretation and utilisation’ of the framework.

### Interpretation and utilisation

#### International symposium- eminence

Two invited experts (external to DEDIPAC) in migrant health gave their views about the draft concept map, particularly where there were gaps in published literature and key research challenges for the future. This was followed by a short interactive discussion drawing on experiences and views from the audience (*n* = 44), during which, ideas were captured from symposium participants. The discussion resulted in the identification of a further 13 factors (6 for diet, 7 for physical activity), which were subsequently incorporated into the framework. This process led to a summary of the research challenges and knowledge gaps identified (Fig. 1- step 6).

#### Finalise systems-based framework

The rating of individual factors and clusters were assembled for the separate diet and physical activity frameworks. A face-to-face meeting of the DEDIPAC ethnic minority and general population teams was held to discuss the integrated framework (Fig. 1- step 7) and consensus was sought at the cluster level. It was the final step in the framework development process to compare the scoring of factors and ranking of clusters across minority ethnic populations with the general European population and to discuss the implications for research and interventions of the factors and clusters in the final framework.

Frameworks in the general population of factors influencing physical activity [29] and sedentary behaviour [30] separately, as well as for dietary behaviour [28] were developed in parallel by the general population teams in the DEDIPAC-KH. While the factors for ethnic minority populations fed into these general population frameworks, they were developed in separate processes [28–30]. This allowed for a post-hoc comparison of clusters identified in the general population with those identified in the ethnic minority framework.

## Results

### Emerging clusters for dietary and physical activity behaviours

Seven distinct clusters (containing 85 factors) were identified for dietary behaviour of ethnic minority populations (Additional file 1: Table S1): ‘migration context’; ‘social and cultural environment’; ‘food beliefs and perceptions’; ‘accessibility of food’; ‘the body’; ‘psychosocial’; and ‘social and material resources’. The highest number of factors were identified in the ‘social and cultural environment’ cluster (20 factors), followed by ‘food beliefs and perceptions’ (13 factors). The ‘psychosocial’ and ‘accessibility of foods’ clusters had an equal number of factors (12 factors each). Only five factors were identified for ‘the body’ cluster.

Eight distinct clusters (containing 183 factors) were identified for physical activity behaviours (Additional file 1: Table S2): health and health communication; political environment; social and cultural environment; psychosocial; institutional environment; physical environment and opportunity; social and material resources; and migration context. The highest number of factors were identified in the social and cultural environment cluster (53 factors), followed by the psychosocial cluster (38 factors). Whilst the lowest number was identified for the political environment cluster (3 factors).

### Priority ranking of factors for dietary behaviour

One-third of the top rated 15 factors for diet were related to food accessibility (Table 2). These factors scored highly in all criteria; and three of these factors (food availability, food policy, food price) were in the top five overall; indeed food price scored highest for its likely impact on population behaviour.

**Table 2:** Ranking of top 15 dietary factors (presented in table are mean (SD))

Rank^a^	Factor	Modifiability	Priority for research	Effect on Behaviour	SUM	Cluster name
1	Food availability	3.37 (1.18)	3.50 (1.00)	3.93 (1.10)	10.80	Accessibility of food
2	Food policy	3.27 (0.96)	3.95 (0.94)	3.57 (1.19)	10.78	Accessibility of food
3	Perceived barriers	3.82 (0.75)	3.43 (1.27)	3.52 (0.99)	10.77	Psychosocial
4	Nutrition knowledge	4.23 (0.73)	3.20 (1.20)	3.22 (0.89)	10.65	Resources/social capital
5	Food prices	2.68 (1.33)	3.72 (1.17)	4.20 (0.77)	10.60	Accessibility of food
6	Children’s food preferences	3.43 (0.75)	3.35 (0.93)	3.72 (0.91)	10.50	Food beliefs & perceptions
7	Food-related life-style	2.95 (1.00)	3.70 (0.98)	3.77 (1.11)	10.42	Accessibility of food
8	Social role of food	2.23 (0.88)	3.85 (0.81)	3.98 (0.80)	10.07	Food beliefs & perceptions
9	Psycho-social stress	2.95 (1.00)	3.65 (1.09)	3.45 (0.94)	10.05	Psychosocial
10	Food beliefs	2.93 (0.81)	3.42 (0.82)	3.68 (0.92)	10.03	Food beliefs & perceptions
11	Social networks	2.55 (0.83)	3.62 (0.59)	3.68 (0.73)	9.85	Social and cultural
12	Subjective norms	2.87 (1.05)	3.33 (0.87)	3.52 (0.94)	9.72	Psychosocial
13	Accessibility of traditional foods	3.32 (1.08)	3.08 (1.06)	3.05 (1.19)	9.45	Accessibility of food
14	Immigrant related policy	3.05 (1.05)	3.52 (1.14)	2.85 (1.18)	9.42	Migration context
15	Level of acculturation	2.62 (1.20)	3.32 (0.98)	3.28 (0.91)	9.22	Social and cultural

All scores on a scale of 1–5, with 5 as most modifiable, strongest effect, largest priority

^a^Position that the factors were ranked in from the 79 diet factors based on 3 criteria of ‘research priority’, ‘expected modifiability’ and ‘potential effect size on behaviour’; factors were scored by 20 people for all 3 criteria

The factors in the ‘food beliefs and perceptions’ cluster that scored highly related to children’s food preferences and the social role of food and food beliefs; although the latter two did not score well for modifiability, as they were seen as difficult to change, presumably because of their socially engrained nature. The psychosocial cluster of factors that reached the top 15 were related to perceived barriers, psychosocial stress and subjective norms influencing dietary behaviours. Perceived barriers in particular scored well for modifiability, suggesting barriers could be targeted in subsequent interventions. Even though so many factors emerged in the social and cultural cluster (Additional file 1: Table S1), only two of these were ranked highly enough across all criteria to be included in the final list of 15 factors (social networks; level of acculturation).

Only ‘nutrition knowledge’ scored well amongst the ‘material and social resources’ cluster (Table 2); the other factors in this cluster scored low on modifiability and therefore were not seen as imperative to study, e.g. income. Only ‘immigrant related policy’ in the host country scored well amongst the ‘migration context’ cluster, but even then it did not score well as a research priority. Others in this cluster had low scores for modifiability, such as the political context in the host country. No factors in ‘the body’ cluster emerged in the top 15.

### Priority ranking of factors for physical activity

Almost one-third of the top rated 15 physical activity factors were related to the ‘physical environment and opportunity’ cluster (Table 3). Two factors related to provision of culturally sensitive and/or women only facilities (Table 3). Four psychosocial factors were ranked highly overall, scoring well for modifiability (knowledge of physical activity, lack of physical activity skills, expectations of physical activity, attitudes).

**Table 3:** Ranking of top 15 factors related to physical activity behaviours (presented in table are mean (SD))

Rank^a^	Factor	Modifiability	Priority for research	Effect on Behaviour	SUM	Cluster name
1	Lack of physical activity at school	3.82 (1.10)	3.53 (0.86)	3.67 (0.77)	11.02	Institutional environment
2	Knowledge of physical activity	4.11 (0.96)	3.08 (1.37)	3.15 (1.10)	10.35	Psychosocial
3	Social influence	2.86 (1.02)	3.60 (1.09)	3.78 (0.88)	10.25	Social and cultural environment
4	Lack of physical activity skills	3.61 (1.04)	3.14 (0.86)	3.39 (0.98)	10.14	Psychosocial
5	Parental attitudes	3.10 (0.68)	3.38 (1.09)	3.61 (0.92)	10.08	Social and cultural environment
6	Lack of culturally sensitive facilities	3.28 (1.07)	3.44 (0.92)	3.33 (0.84)	10.05	Physical environment and opportunity
7	Expectations of physical activity	3.33 (1.08)	3.22 (1.06)	3.45 (1.10)	10.00	Psychosocial
8	Attitudes	3.38 (0.98)	3.27 (1.07)	3.35 (1.03)	10.00	Psychosocial
9	Facilities available	3.05 (1.06)	3.38 (0.78)	3.50 (0.96)	9.94	Physical environment and opportunity
10	Access to a play area	3.23 (0.94)	3.26 (0.89)	3.44 (0.78)	9.93	Physical environment and opportunity
11	Lack of knowledge of host culture	3.72 (0.96)	2.94 (0.87)	3.12 (0.68)	9.78	Migration context
12	Area deprivation	2.50 (0.86)	3.50 (0.92)	3.72 (0.83)	9.71	Social and material resources
13	Lack of women only facilities	3.25 (1.00)	3.20 (1.11)	3.22 (1.00)	9.66	Physical environment and opportunity
14	Primary health care	3.36 (1.14)	3.23 (1.11)	3.05 (1.00)	9.64	Health and health communication
15	Habitus	2.60 (1.14)	3.23 (1.40)	3.63 (1.29)	9.46	Social and cultural environment

All scores on a scale of 1–5, with 5 as most modifiable, strongest effect, largest priority

^a^Position that the factors were ranked in from the 183 Physical activity/Sedentary behaviour factors based on the overall scores of the 3 criteria of ‘research priority’, ‘expected modifiability’ and ‘potential effect size on behaviour’; factors were scored by 20 people for all 3 criteria

‘Physical activity at school’ was ranked first, performing well in terms of its likely impact on population health, due to the potential reach that school based interventions can have, suggesting schools could be a priority setting in subsequent interventions. Only ‘area deprivation’ scored highly amongst the ‘material and social resources’ cluster, as it was seen to have an important impact on behaviours, but it scored less well for modifiability. Other factors related to material and social resources, such as income, scored less well, as they were seen as hard to modify.

Only three factors ranked highly (Table 2) amongst the 53 factors that emerged in the ‘Social and cultural environment’ cluster (Additional file 1: Table S2). Two of these appeared closely inter-related (social influence, habitus), where habitus was seen as how individuals perceive and react to the social world around them. The third factor (parental attitudes) reflects the high ranking given to children’s physical activity behaviours. Of the 12 factors in the ‘health and health communication’ cluster, only ‘primary health care’ was ranked highly. The high rating of primary health care in part stems from the central role of health professionals conveying the importance of physical activity, as it was seen as modifiable, with potential to reach many people through interventions. Only ‘lack of knowledge of host culture’ scored well in ‘migration status’, as it was seen as modifiable, whereas the other factors in this cluster were perceived as difficult to change. No factors in ‘the political environment’ emerged amongst those in the top 15.

### Cluster ranking for factors influencing dietary and physical activity behaviours

For both diet and physical activity, ‘psychosocial’ factors was the top ranking cluster, which scored highly on all three criteria, but particularly as a priority for research and its likely impact on behaviour (Table 4). The scores for the remaining clusters were ranked closely behind. The ‘migration context’ cluster of factors scored lowest, mainly because it had a low score for modifiability. Social and material resources were seen to have an important impact on behaviour, but scored less for modifiability. Overall, the relatively close ranking of the clusters for both behaviours suggested that all could have a part to play in developing interventions and research.

**Table 4:** Ranking of clusters for dietary and physical activity behaviour factors

Cluster	Number of factors^a^	Mean Modifiability max score 5	Mean Priority for research max score 5	Mean effect on behaviour max score 5	SUM of raw scores	Mean score/factor^b^ max score 15	Cluster rank^c^
Dietary behaviour							
Psychosocial	12	3.07	3.10	3.69	108.43	9.04	1
Food beliefs and perceptions	11	2.73	2.88	3.34	98.50	8.96	2
Social and material resources	10	2.36	3.04	3.51	89.03	8.90	3
Accessibility of food	12	2.46	3.02	3.31	105.57	8.80	4
The body	5	2.87	2.54	3.14	42.75	8.55	5
Social and cultural environment	18	1.99	2.84	3.19	144.27	8.02	6
Migration context	13	1.47	2.51	3.08	91.70	7.05	7
Physical activity behaviours							
Psychosocial	38	2.70	2.64	3.17	323.62	8.52	1
Institutional environment	14	2.68	2.29	3.00	114.73	8.20	2
Political environment	3	2.14	2.95	3.05	24.44	8.15	3
Social and cultural environment	49	2.13	2.64	3.12	394.32	8.05	4
Physical environment and opportunity	31	2.45	2.47	3.08	247.81	7.99	5
Social and material resources	12	1.83	2.66	3.41	94.71	7.89	6
Health and health communication	12	2.29	2.35	3.22	94.34	7.86	7
Migration context	17	1.76	2.31	2.70	121.90	7.17	8

^a^The number of factors here does not include those 6 factors identified at the ISBNPA symposium, as they were not scored. The full list is in Tables 1 and 3

^b^Mean is the total sum divided by the number of factors- so a maximum of 15 could be gained as 3 criteria on a Likert scale of 1–5, with 5 as most modifiable, strongest effect, largest priority

^c^Position that the factors were ranked in based on overall SUM/number of factors in each cluster

### A systems-based framework – A tool for prioritising research and interventions

**Fig. 2:**
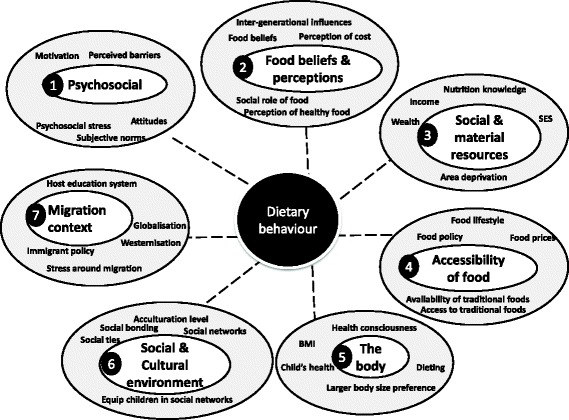
A systems-based framework of the priority factors and clusters influencing dietary behaviours in ethnic minority populations living in Europe. The numbers represent the order of ranking of the clusters. The factors listed under each cluster are the top 5 factors ranked by a range of experts from a wide array of disciplines for their importance based on 3 criteria of ‘research priority’, ‘expected modifiability’ and ‘potential effect size on behaviour’. Dotted lines indicate that factors in the cluster are associated with behaviours, but they do not indicate evidence for causation

**Fig. 3:**
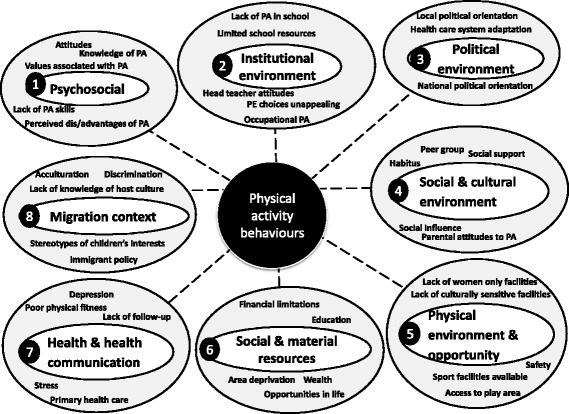
A systems-based framework of the priority factors and clusters influencing physical activity behaviours in ethnic minority populations living in Europe. The numbers represent the order of ranking of the clusters. The factors listed under each cluster are the top 5 factors ranked by a range of experts from a wide array of disciplines for their importance based on 3 criteria of ‘research priority’, ‘expected modifiability’ and ‘potential effect size on behaviour’. Dotted lines indicate that factors in the cluster are associated with behaviours, but they do not indicate evidence for causation

Figures 2 and 3 summarise the clusters that emerged and their priority ranking. The top scoring five factors in each cluster are highlighted.

**Fig. 4:**
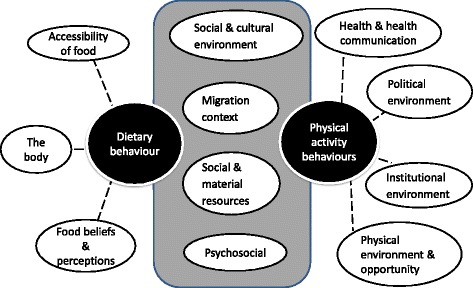
A systems-based integrated framework of the clusters influencing dietary and physical activity behaviours in ethnic minority populations living in Europe. Dotted lines indicate that factors in the cluster are associated with behaviours, but they do not indicate evidence for causation

An integrated framework for the major clusters of factors influencing both dietary and physical activity behaviours and the overlap between them is illustrated in the overall framework (Fig. 4).

The clusters of factors influencing the different behaviours were integrated to illustrate both similar and distinct clusters of factors. Four of the clusters were similar (psychosocial; social and cultural; social and material resources; and migration context) for diet and physical activity behaviours.

### Research priorities for ethnic minorities compared with those for the general host population

Some similarities and differences were observed between ethnic minorities and general host populations. Similar sub-categories of clusters of factors emerged in the general population frameworks for diet [28], physical activity [29] and sedentary behaviours [30] combined, except for those in the migration context (Table 5).

**Table 5:** Comparing emerging clusters and their overall ranking in ethnic minorities with sub-categories in the general European population for dietary and physical activity behaviours

Dietary behaviours			Physical activity behaviours			
	Cluster Rank			Cluster Rank		
Cluster name	Ethnic minorities	General population^a^	Cluster name	Ethnic minorities (PA/SB)	General population (PA)^b^	General population (SB)^c^
Psychosocial	1	7	Psychosocial	1	3	2
Food beliefs and perceptions	2	17	Institutional environment	2	–	1
Social and material resources	3	18	Political environment	3	5	6
Accessibility of food	4	2	Social and cultural environment	4	2 and 4	5
The body	5	30	Physical environment and opportunity	5	1	3
Social and cultural environment	6	20	Social and material resources	6	6	**–**
Migration context	7	–	Health and health communication	7	–	4
			Migration context	8	–	**–**

Matches here are based on overlap of the individual factors included in each sub-category^a^ or cluster but it should be noted that overlap may only be partial. All scores were ranked based on criteria including priority for research, modifiability and population-level effect (and relationship strength^a^)

There are a total of 51 sub-categories in the general population diet framework^a^ [31], 6 clusters in both the physical activity framework^b^ [32] and sedentary behaviour framework^c^ [33]

[PA = physical activity and SB = sedentary behaviour]

The clusters of ‘health and health communication’ and ‘institutional environment’ did not emerge in the general population for physical activity; and ‘material and social resources’ did not emerge as a distinct cluster of factors influencing sedentary behaviours in the general population.

The ‘social and cultural’ cluster was ranked first for overall importance for its influence on physical activity amongst ethnic minority populations. Even though social and cultural factors emerged in the general population, the overall cluster was ranked lower for physical activity and sedentary behaviour (Table 5). The importance of psychosocial factors in the general population was ranked slightly less than for ethnic minority groups, as they were ranked in second and third position for sedentary behaviour and physical activity respectively. The political environment was ranked higher for its importance in influencing physical activity in ethnic minority populations than in the general population, where it was ranked last (sedentary behaviour) or next to last (physical activity) (Table 5).

For diet, the importance of ‘psychosocial’ factors, ‘food beliefs and perceptions’, ‘social and material resources’, and the ‘social and cultural environment’ were all ranked lower in the general population than for ethnic minority groups (Table 5). However, the importance of the cluster of factors relating to the ‘accessibility of food’ emerged as important across all populations.

## Discussion

A system-based framework was established in this study to summarise the factors influencing dietary and physical activity behaviours in ethnic minority populations living in Europe. This is the first framework developed using a formal consensus methodology, drawing upon wide transdisciplinary evidence and eminence. It is envisaged that the framework will primarily be used as a tool to stimulate operationalisation and contextualisation for research and interventions.

There was insufficient evidence from specific ethnic minority groups, so therefore they were treated together, as there are shared experiences in the lives of people from ethnic minority populations that justify grouping them together. However, as with majority host populations, it is important to acknowledge the heterogeneity of ethnic minority populations living in Europe [36]. The different clusters are likely to interact, implying that factors in a specific cluster operate differently, depending on the factors in other clusters. In addition, clusters are highly dynamic, and might change over time as populations evolve, as the needs of ethnic minority populations are not static.

### Implications for *research priorities* for ethnic minority populations

The framework highlights key research priorities for ethnic minority populations. For instance, addressing the highest rated factors associated with dietary behaviour would involve consideration of several clusters including ‘accessibility of food’, ‘psychosocial’ and ‘resources/social capital’. The clustering of factors in this way might precipitate a shift in the way complex behaviours are viewed; from a simple approach focusing on individual level factors, to a more holistic systems approach. In our study, psychosocial factors were ranked highly overall, particularly as they were seen as modifiable, which probably explains why research and interventions tend to focus on individual psychosocial factors [26].

The lack of evidence to attribute causation and effect strength is a major gap and research on causal models and pathways needs developing. Identifying and visualising inter-connections between factors remains difficult to do without data on their relationships, which requires more research taking a systems approach [22, 24, 26]. Both qualitative and longitudinal quantitative research provide useful insights for these inter-linkages and pathways that lead to dietary and physical activity behaviours [36, 40].

Practical considerations include the need for multidisciplinary researchers involved in understanding and changing behaviours to develop skills to evaluate the impact of complex, upstream, population-level interventions on the underlying clusters of factors [26], as well developing skills in cultural adaptation. Furthermore, in light of the peak migration in 2015–16 to Europe [41], new research regarding the impact of dietary behaviours on health for these populations is required, particularly amongst vulnerable migrant populations, including refugees, unaccompanied children and illegal migrants.

Research on ethnic minority populations often look through a lens of difference [36], i.e. focussing on what is different rather than similar with the general population, which may explain the wealth of social and cultural factors identified through this process. Although important, there is also scope for investigating commonalities, for example, how factors that drive dietary and physical activity behaviours in the majority population influence these behaviours amongst ethnic minority populations, which emphasises the needs for a systems approach across all populations. For example, one-third of the top rated 15 factors for diet were related to food accessibility, including food availability, how food policy shapes access to food and the price of food, which all emerged as important areas for research for the general population too. Interventions targeting these factors will require a whole population approach.

Most of the factors specific to the ‘migration context’ were not seen as a research priority, possibly because they would require studies involving several countries to research populations in different countries, and the context was seen as hard to change. The converse is also true, as research in the majority population seldom sheds light on how social and cultural factors influence behaviours, which may explain why they were ranked lower in the general population framework. The importance of research on collective behaviours, especially on the social practices that shape social habits and therefore practices around diet and physical activity is key for all population groups [40, 42], regardless of their ethnicity.

### Implications for developing *interventions* for ethnic minority populations

The study’s findings have highlighted that there are unlikely to be quick fixes or tipping points that can be isolated to change behaviours, rather, several factors could be targeted to improve diet and/or physical activity behaviours across the inter-related system of clusters. This reinforces the need for a systems approach in planning multi-faceted interventions in order to account for the (sometimes unexpected) interaction between the factors influencing behaviour. Conventional approaches focusing on individual level behaviour change are insufficient [26].

The contextualisation for interventions is crucial too, which is in contrast with the high priority given to the individual level psychosocial factors in this study. This finding is largely a reflection of prevailing perceptions that these are easy to change. However, changing individual level factors, whilst the context remains the same, is insufficient to drive behaviour change, in view of the socially and culturally embedded nature of dietary [43] and physical activity behaviours.

This study’s findings have highlighted much commonality between the factors influencing the behaviours in ethnic minority groups and the majority population. This begs the question of whether interventions are needed that address factors that are specific to ethnic minority groups, or whether mainstream interventions should be encouraged that can reach all groups. The study suggests that ‘mainstream’ interventions targeting the general population could address many factors identified as there was much in common between minority and host populations, such as food policy, food pricing, physical activity at school, access to play areas, area-level deprivation and so forth. However, even if factors are shared, their importance and focus might differ, e.g. the need for women only facilities, the social role of food might be stronger in more collective cultures. There are specific factors in the context of migration that will need to be addressed at a higher policy level, including policies encouraging integration.

Two different approaches for developing interventions that can reach ethnic minority populations are advocated for [44]. These consist of either adapting mainstream interventions for the majority population to be ‘diversity sensitive’, so that they can be equally effective for all citizens regardless of their cultural, religious or ethnic background, or alternatively developing ‘migrant-specific’ interventions by culturally adapting services and interventions to individual backgrounds of specific minority ethnic groups. The framework developed could be used to develop either approach, as well as encourage new approaches. Most of the evaluations of culturally sensitive interventions have been conducted in the US [45, 46] and may not be transferable. Additionally, evidence has indicated that evaluations lack explicit information about the components of cultural adaptation, and little or no detail is provided regarding how interventions are cultural adapted [47]. Evaluating interventions in a way that goes beyond ‘what works’, but also identifies ‘for whom it works and in what context’, such as realist approaches [48] would be well adapted to unravel the underlying processes. It should also be emphasised that this framework does not provide ready-made answers for intervention development. As for the case for ‘majority’ populations, a needs assessment will remain a necessary part of the process [49].

### Methodological limitations

An important limitation was that whilst the frameworks for physical activity and sedentary behaviour followed a very similar concept mapping approach to determining clusters of factors, the framework for dietary behaviours did not include emerging clusters [28]. The dietary behaviour framework was developed by sorting individual factors into pre-defined categories with a positivist top-down process, using a socio-ecological approach [31]. Whereas a constructivist approach was taken in the development of the other frameworks, as clusters emerged from the data. However, both approaches are holistic, given that the clusters that emerged were so similar with the different approaches. In addition, factors were rated in a different manner in the general population’s dietary behaviour framework as the overall priority for research was based on a weighted average of ratings for their modifiability, relationship strength and population-level effect [28].

Another limitation is that research participants did not cluster factors as individuals, but collectively as a group, meaning that clusters could not be created mathematically using cluster analysis [32]. This approach was decided against due to time limitations during data collection for individuals to cluster factors together separately. Even in the case of individual clustering, the results of cluster analysis often require visible adjustment to make them meaningful [32].

The decision to only request scoring of the top 15 ranking factors in the international symposium may have introduced bias, as it is unknown if these factors would be different if other people from within the broader DEDIPAC-KH or from outside it had participated in the rating of all factors. However, new/additional factors were explicitly sought from external participants, in order to compensate for this limitation.

The concept mapping exercise led to a hypothesised ranking of factors within clusters, while the systems approach implies that the interaction of context and inter-related factors is what influences behaviour [26]. However, evidence for links between factors was not found, due to a lack of research on the underlying mechanisms. Given this, there remains uncertainty about the specific factors that are a priority for research. A further limitation was the profile of participants. The framework was informed by academic researchers and although some have extensive experience with ethnic minority and migrant origin populations, input from individuals from agencies who work closely with different populations would have improved its completeness. Academic specialists in migrant health were invited as experts, enhancing confidence in the potential utility of the framework presented.

Other limitations included the lack of research highlighting the drivers of dietary and physical activity behaviours across the life-course, as most research targeted adults. There were insufficient studies to differentiate by age or life-course and further research is particularly required on children and adolescents, as they clearly have a role in influencing behaviours of the whole family. There was limited research on sedentary behaviour among ethnic minorities. Some ethnic minority groups were under-represented, particularly recent migrant populations to Europe, which should also be a priority for research.

## Conclusions

This is the first systems-based framework to be developed that sheds light on dietary and physical activity behaviours of ethnic minority populations living in Europe, drawing on both evidence and eminence. Distinct clusters emerged for both dietary and physical activity behaviours, of which four clusters were similar across behaviours (social and cultural environment; social and material resources; psychosocial; and migration context), suggesting that an integrated approach for interventions across behaviours in these clusters of factors could bring maximum benefit. Similar clusters of factors emerged in the majority population frameworks for diet and physical activity behaviours, but their relative importance differed for ethnic minority populations, compared with the majority population.

Our findings illustrate that innovative research and interventions need to be developed that are sensitive to the needs of ethnic minority populations. Dietary and physical activity behaviours are intransigent and addressing them will require enormous innovation. A systems approach may help in shifting the current public health paradigm towards a more holistic approach that considers what works for whom and in what context, in order to ensure that ethnic minorities are included, alongside mainstream European populations.

## Additional file

Additional file 1: Table S1. Concept map of the 85 factors and 7 clusters that emerged influencing dietary behaviours in ethnic minority groups. **Table S2.** Concept map of the 183 factors and the 8 clusters that emerged influencing physical activity behaviours (DOCX 46 kb)

## Abbreviations

BMI: Body mass index
DEDIPAC: DEterminants of DIet and Physical ACtivity
PA: Physical activity
SB: Sedentary behaviour
SES: Socioeconomic status
