# Supplementary material for: Developing a systems-based framework of the factors influencing dietary and physical activity behaviours in ethnic minority populations living in Europe - a DEDIPAC study
Source: Int J Behav Nutr Phys Act. 2017 Nov 7;14:154. doi: 10.1186/s12966-017-0608-6 (PMC5678802; doi:10.1186/s12966-017-0608-6)
Supplement: Additional file 1: Table S1. — Concept map of the 85 factors and 7 clusters that emerged influencing dietary behaviours in ethnic minority groups. Table S2. Concept map of the 183 factors and the 8 clusters that emerged influencing physical activity behaviours (DOCX 46 kb) [file 12966_2017_608_MOESM1_ESM.docx]

**[Additional file](http://www.ijbnpa.org/authors/instructions/research#preparing-additional-files) 1**

| **Table S1: Concept map of the 85 factors and 7 clusters that emerged influencing dietary behaviours in ethnic minority groups** | | | | | | | | | | | | | | | | | | | | | |
| --- | --- | --- | --- | --- | --- | --- | --- | --- | --- | --- | --- | --- | --- | --- | --- | --- | --- | --- | --- | --- | --- |
|  | | **Migration context** | | **Social & cultural environment** | | **Food beliefs & perceptions** | | | | **Accessibility of food** | | | **The body** | | | **Psychosocial** | | | **Social & material resources** | | |
| **No. of factors** | | 13 | | 20 | | 13 | | | | 12 | | | 5 | | | 12 | | | 10 | | |
| **Factors** | | Westernisation  Region of origin  Urban or rural dweller  Age at migration  Country of birth  Length of stay in host country  Place of residence in host country  Education before migration^1^  Host education system^1^  Stress around migration^1^  Globalisation^1^  Historical and geographical origin^1^  Immigration policy^2^ | | Cultural identity  Ethnic identity  Ethnicity  Religious beliefs  Equip children in social networks  Perception of host culture  Acculturation level  Religious prescriptions  Socialization process in place of residence  Conformity to tradition  Traditional dietary values/beliefs  Gender  Age  Social networks  Social ties  Social bonding  Women’s caring role^1^  Penetration of ethnic food in host country^2^  Social value of ethnic food in host country^2^  Collectivist norms^2^ | | Status of traditional vs convenience foods/diets  Familiarization of host foods before migration  Familiarization with host country foods  Husband's food preferences  Children's food preferences  Inter-generational influences on diet  Parental dietary habits  Perception of healthy food  Food beliefs  Perception of cost  Social role of food  Who is the food decision maker^2^  Lack of knowledge to make traditional foods healthier^2^ | | | | Availability of traditional foods  Accessibility of traditional foods  Food prices  Food-related lifestyle  Neighbourhood level physical proximity  Season  Family’s neighbourhood (ethnic enclave)  Lack of time for cooking traditional foods  Time for food preparation  Change in lifestyle (work/school commitments)  Food policy^1^  Work-life balance^1^ | | | Health consciousness  Dieting  BMI  Larger body size preferences  Child’s health | | | Taste preferences  Attitudes  Subjective norms  Perceived behaviour control  Perceived behavioural intention  Perceived group norms  Past behaviour  Motivation  Food neophobia  Psychosocial stress^1^  Perceived barriers^1^  Time discounting^1^ | | | Competency in host language  Educational attainment  SES  Income  Nutrition knowledge  Social class^1^  Area deprivation^1^  Opportunities in life^1^  Adopt behaviour of community^1^  Wealth^1^ | | |
| 63 factors emerged from the systematic mapping review [36] and 16 extra factors^1^ identified from eminence at brainstorming step and 6 factors^2^ identified at international symposium). | | | | | | | | | | | | | | | | | | | | | |
| **Table S2: Concept map of the 183 factors and the 8 clusters that emerged influencing physical activity behaviours** | | | | | | | | | | | | | | | | | | | |  |  |
|  | **Health & health communication** | | **Political environment** | | **Social & cultural environment** | | | **Psychosocial** | **Institutional environment** | | | **Physical environment & opportunity** | | | **Social & material resources** | | | **Migration context** | | |  |
| **No. of factors** | 12 | | 3 | | 53 | | 38 | | 15 | | 32 | | | 12 | | | 18 | | | |  |
| **Factors** | Primary health care  Poor physical fitness  Stress  Depression  Lack of follow-up  Healthcare support  Health conditions  Mental health  Pain  Physical health  Bad health  Religious fasting | | Local Political orientation  National political orientation  Health care system adaptation | | Habitus  Social influence  Parental attitudes to PA  Social support  Peer group  Social resources  Social network  Social environment  Activities in own community  Shame  Cultural requirements  Preferred mode of transportation  Women as caregiver/mother  Car use  Attitude of peers  Purposeful PA selfish  Gym based exercise unfamiliar  Lack of 'exercise culture'  Structural constraints in family  Collectivist norms  Religious community  Functional support  Social acceptance of PA  Women not to be alone outside  Behaviour of others  Few active friends or family  Ethnic minority concentration  Family  PA role models  Language of PA  Inactive parental lifestyle  Parents participation in organised sports  Commitments within family  Overprotective family  PA irrelevant to disease  Gender  Religion and culture  Concepts of aging/generation  Work ethics  Ethnic group  Traditional power relations  Facilitative relatives  Modesty  Religious requirements  Religious festivals  Religious prayer  Traditional authorities  Parental marital status  Work-life balance^1^  Adopt behaviour of neighbours^2^  Knowledge of games/rules for sport^2^  Cultural norms about sitting^2^  Pressure to do well academically^2^ | | Attitudes  Values associated with PA  Lack of PA skills  Knowledge of PA  Perceived dis-/advantages of PA  Lack of enjoyment of PA  Lack of PA routine  PA as part of everyday life  Self-efficacy  Lack of knowledge of area  Ideas of ideal body  Confidence level  Health beliefs about PA  Interest in PA  Habit  Behavioural control  Views on age, lifestyle and health  Perceived restrictions  Notions of leisure-time PA  Lack of intention  Motivation  Self-image  Ability to use health care  Concerns about safety  Goal setting  Not the sporty type  Fear of falling  Walking to school resisted  Experience of PA/PE  Want to be fit  Dangers of environments and strangers  Preference of PA  Identity  Fear of racism  Prevent disease  Karma/fatalism  Not gaining weight  Psychosocial stress^1^ | | Lack of PA in school  Limited school resources  Occupational PA  PE choices unappealing  Head teacher’s attitude and resources  Lack of separation work-leisure  Demands of curriculum  Exam pressure  School  Priorities in school  Lack of extra-curricular staff  Negotiating participation  Emphasis on competitive sports  Traditional livelihood  Workplace policy for PA^2^ | | Lack of culturally sensitive facilities  Sports facilities available  Access to play area  Lack of women only facilities  Physical environment  Convenience of SB, e.g. TV  Logistics of activities  Lack of appropriate activities  Expenses  Lack of information  Lack of green space  Few sidewalks  Lack of open space  Lack of safe storage in school for bikes/kit  Unattractive neighbourhood  Transportation and infrastructure  Crime  Lack of gym instructor support  Home appliances limit PA  Living in urban area  Media portrayal of unsafe environment  Too much traffic  Financial incentives  Religious dress requirements  Short-term activities  Small housing  Financial sanctions  Cold  Bad weather  Climate  Safety^1^  Public transport policy^2^ | | | Financial limitations  Education  Time constraints  Occupation  Income  Practicalities/responsibilities  Parental education level  Parental employment  Opportunities in life^1^  Social class^1^  Area deprivation^1^  Wealth and income^1^ | | | Lack of knowledge of host culture  Acculturation  Discrimination  Stereotypes of children’s interests  Racial harassment  Social ties with home country  Migration  Type of minority status  Country of birth  Immigrant parent  Time since migration  Immigration history  Age at time of migration  Globalisation^1^  Education system before migration^1^  Immigrant policy^1^  Stress around migration^1^  Integration policy^2^ | | | |  |
|  | 165 factors emerged from the systematic mapping review [37] and 11 extra factors^1^ were identified at brainstorming step and 7 factors^2^ identified at international symposium | | | | | | | | | | | | | | | | | | | |  |
